# Metabolic liver function measured *in vivo* by dynamic ^18^F-FDGal PET/CT without arterial blood sampling

**DOI:** 10.1186/s13550-015-0110-6

**Published:** 2015-05-14

**Authors:** Jacob Horsager, Ole Lajord Munk, Michael Sørensen

**Affiliations:** Department of Nuclear Medicine & PET Centre, Aarhus University Hospital, DK-8000 Aarhus, Denmark; Department of Hepatology & Gastroenterology, Aarhus University Hospital, Noerrebrogade 44, DK-8000 Aarhus C, Denmark

**Keywords:** Dynamic PET/CT, Metabolic liver function, Galactose, Non-invasive, Image-derived input

## Abstract

**Background:**

Metabolic liver function can be measured by dynamic PET/CT with the radio-labelled galactose-analogue 2-[^18^F]fluoro-2-deoxy-D-galactose (^18^F-FDGal) in terms of hepatic systemic clearance of ^18^F-FDGal (*K*, ml blood/ml liver tissue/min). The method requires arterial blood sampling from a radial artery (arterial input function), and the aim of this study was to develop a method for extracting an image-derived, non-invasive input function from a volume of interest (VOI).

**Methods:**

Dynamic ^18^F-FDGal PET/CT data from 16 subjects without liver disease (healthy subjects) and 16 patients with liver cirrhosis were included in the study. Five different input VOIs were tested: four in the abdominal aorta and one in the left ventricle of the heart. Arterial input function from manual blood sampling was available for all subjects. K*-values were calculated using time-activity curves (TACs) from each VOI as input and compared to the *K*-value calculated using arterial blood samples as input. Each input VOI was tested on PET data reconstructed with and without resolution modelling.

**Results:**

All five image-derived input VOIs yielded K*-values that correlated significantly with *K* calculated using arterial blood samples. Furthermore, TACs from two different VOIs yielded K*-values that did not statistically deviate from *K* calculated using arterial blood samples. A semicircle drawn in the posterior part of the abdominal aorta was the only VOI that was successful for both healthy subjects and patients as well as for PET data reconstructed with and without resolution modelling.

**Conclusions:**

Metabolic liver function using ^18^F-FDGal PET/CT can be measured without arterial blood samples by using input data from a semicircle VOI drawn in the posterior part of the abdominal aorta.

## Background

Dynamic PET/CT with the tracer 2-[^18^F]fluoro-2-deoxy-D-galactose (^18^F-FDGal) enables *in vivo* measurement of metabolic liver function in terms of hepatic systemic clearance of ^18^F-FDGal (*K*, ml blood/ml liver tissue/min) [1-3]. In short, the procedure includes intravenous bolus administration of 100 MBq ^18^F-FDGal in the beginning of a 20-min dynamic PET recording of the liver with arterial blood samples from a catheter in a radial artery. *K* is then calculated using a kinetic model of irreversible trapping of ^18^F-FDGal-1-phosphate in the liver using the time-activity curve (TAC) from arterial blood (Artery-TAC) as input function and the TAC from liver tissue (Liver-TAC) as output function [1-3]. The use of single arterial input concentration instead of the mixed porto-arterial concentration to the liver was validated in pigs [3], and the final protocol using 20-min PET recording was validated in patients with cirrhosis and healthy subjects [1,2]. The rate-limiting step in hepatic metabolism of ^18^F-FDGal is phosphorylation of the tracer by galactokinase to ^18^F-FDGal-1-phosphate. *K* of ^18^F-FDGal is flow-independent and depends only on enzymatic capacity, i.e., *V*_max_ of galactokinase [1,2], and it thus reflects metabolic function.

^18^F-FDGal PET/CT enables creation of three-dimensional images of metabolic liver function which was used to demonstrate that patients with cirrhosis have a lower hepatic metabolism of ^18^F-FDGal compared to healthy subjects in addition to an increased variation in intrahepatic metabolic function [1]. Evaluation of regional differences in metabolic liver function has many potential applications; it could for example be used to spare the best functioning areas of the liver when planning stereotactic radiotherapy of liver tumours [4].

We hypothesized that scan data from the abdominal aorta or the left ventricle of the heart can replace arterial blood sampling, and the aim of the present study was to define an image-derived input-TAC to replace the Artery-TAC for calculating *K* in dynamic ^18^F-FDGal PET/CT studies of metabolic liver function_._ For this purpose, input-TACs from different volumes of interest (VOIs) in the abdominal aorta and left ventricle of the heart were tested. Since image-derived input-TACs are prone to errors caused by spill-over effects from surrounding tissues which may be reduced using images with improved spatial resolution provided by reconstruction algorithms with resolution modelling (point spread function correction) [5], we applied each VOI to PET data reconstructed with and without resolution modelling. Making ^18^F-FDGal PET/CT of metabolic liver function a non-invasive procedure would spare the patient from the discomfort of having a catheter inserted into the radial artery and reduce the radiation burden of the technical staff. The method would thus be safer, simpler, and more general applicable for routine clinical use.

## Methods

Data comprised raw data from two previously published ^18^F-FDGal PET studies: eight patients with cirrhosis [1] and seven subjects with no parenchymal liver disease (healthy subjects) [2]. In addition, unpublished data from nine healthy subjects and eight patients with cirrhosis were included. In total, 16 healthy subjects and 16 patients with cirrhosis were thus included in the study. The study was approved by The Central Denmark Region Committees on Health Research Ethics and conducted in accordance with the Helsinki Declaration.

### PET studies

All subjects were studied in supine position using the same PET/CT camera, a 64-slice Siemens Biograph Truepoint PET/CT camera (Siemens AG, Erlangen, Germany). Before the PET recording, a topogram of the upper abdomen was performed for optimal positioning of the liver within the 21.6-cm transaxial field-of-view of the PET camera followed by a low-dose CT scan (50 effective mAs with CARE Dose4D (Siemens Healthcare, Erlangen, Germany), 120 kV, pitch 0.8, slice thickness 5 mm) for definition of anatomical structures and attenuation correction of PET data. A bolus of 100 MBq of ^18^F-FDGal in 10 ml saline was administered intravenously during the initial 15 to 20 s of a dynamic PET recording (list-mode). In PET recordings exceeding 20 min, only measurements up to 20 min were used in the present study since this is now the standard protocol [1-3]. ^18^F-FDGal was produced in our own radiochemistry laboratory (radiochemical purity ≥ 97%) [6].

All subjects had an Artflon catheter (Artflon, Ohmeda, Swindon, UK) placed percutaneously in a radial artery. During the PET recording, arterial blood samples (0.5 ml) were manually collected for determination of ^18^F-FDGal blood concentrations (kBq/ml) using a well counter (Packard Instruments, Meriden, CT, USA) and corrected for radioactivity decay back to start of the scan.

#### Reconstruction of PET data

Data were reconstructed using two different methods: without resolution modelling (336 matrix, voxel size 2 × 2 × 2 mm^3^, 6 iterations, 21 subsets, 2-mm Gaussian filter, separate prompts/randoms) and with resolution modelling (336 matrix, voxel size 2 × 2 × 2 mm^3^, 4 iterations, 21 subsets, 2-mm Gaussian filter, separate prompts/randoms). Measurements were corrected for radioactivity decay back to start of the scan. Time-frame structure was 18 × 5, 15 × 10, 4 × 30, 4 × 60, and 2 × 300 s, total 20 min.

### Image analysis

#### Liver-VOI

Using fused PET/CT images, regions of interest (ROIs) were drawn in successive image planes in the parenchyma of the right liver lobe avoiding large blood vessels and summed into a volume of interest (Liver-VOI). The same VOI was used to generate a liver time-activity curve (Liver-TAC; liver activity concentration in kBq/ml liver tissue vs. time in minutes) for both reconstruction methods.

#### Aorta-VOI

**Figure 1:**
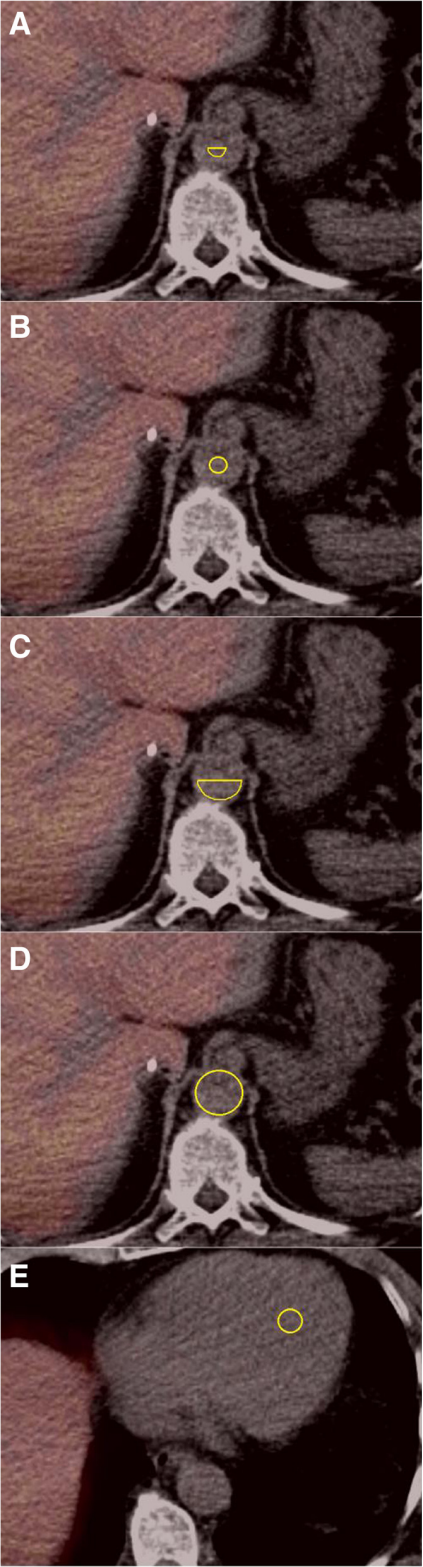
Illustration of the five VOIs tested shown in a transaxial plane. Aorta-VOI-1 **(A)**, Aorta-VOI-2 **(B)**, Aorta-VOI-3 **(C)**, Aorta-VOI-4 **(D)**, and Ventricle-VOI **(E)**.

Four different VOIs in the abdominal aorta were tested; for all four VOIs, ROIs were drawn in adjacent transaxial planes starting 3 planes below the diaphragm (6 mm) and continued for 40 planes in the caudal direction (total length, 8 cm). The ROIs were summed into an Aorta-VOI, and an Aorta-TAC (blood concentration in kBq/ml blood vs. time in minutes) was generated for both reconstruction methods. The four Aorta-VOIs were defined as (Figure 1):

- Aorta-VOI-1: A semicircle drawn in the posterior half of the aorta with the straight line (length one third of the total diameter of the aorta) going through the centre of the vessel. Average volume was 2.95 ml (range, 2.03 to 4.21 ml).
- Aorta-VOI-2: A circular ROI drawn in the centre of the aorta with a diameter of one third of the diameter of the aorta. Average volume was 4.75 ml (range, 3.04 to 6.23 ml).
- Aorta-VOI-3: A semicircle drawn in the posterior half of the aorta with a diameter equal to that of the aorta and with the straight line going through the centre of the vessel. Average volume was 10.23 ml (range, 6.17 to 15.59 ml).
- Aorta-VOI-4: A circular ROI including the entire aorta in each plane included in the VOI. Average volume was 32.1 ml (range, 21.62 to 45.78 ml).

#### Ventricle-VOI

For the VOI in the left ventricle of the heart (Ventricle-VOI), circular ROIs were drawn in four to six adjacent transaxial planes (Figure 1). Average volume was 1.88 ml (range, 0.89 to 5.65 ml).

### Data analysis

Hepatic systemic clearance of ^18^F-FDGal (ml blood/ml liver tissue/min) was calculated according to the Gjedde-Patlak representation of data [7,8] as validated in previous studies [1-3]. In short, the hepatic systemic clearance is defined as the slope of the asymptote fitted to the linear part of the data representation 6 to 20 min after tracer administration [1-3,7,8]. The Liver-TAC was used as output function, and the hepatic systemic clearance of ^18^F-FDGal was calculated for each non-invasive input function (K*) and compared to the value calculated using the Artery-TAC (*K*) which was used as reference value.

### Statistics

Correlation between K* and *K* was tested by the Pearson product–moment correlation coefficient, *r*, and a *p* value < 0.05 was interpreted to indicate a statistically significant correlation. Mean relative deviations of K* from *K*, i.e., (K* − *K*)/*K*, were tested by a one-sample *t*-test, and deviations with a *p* value < 0.05 were considered statistically significant.

## Results

The correlation between K* and *K* was significant for all five VOIs using both reconstruction methods and for both healthy subjects (Table 1) and patients with cirrhosis (Table 2) (*p* < 0.01 for all combinations).

**Table 1:** **Healthy subjects (functional** ^**18**^ **F-FDGal PET/CT of the liver using image-derived non-invasive input function)**

**VOI**	**Without resolution modelling**		**With resolution modelling**	
	**Correlation between** ***K*** * and ***K*** **,** ***r***	**Relative deviation of** ***K*** * from ***K***	**Correlation between** ***K*** * and ***K*** **,** ***r***	**Relative deviation of** ***K*** * from ***K***
Aorta-VOI-1	0.878	−0.044 ± 0.039	0.866	0.016 ± 0.061
	(*p* < 0.01)	(*p* = 0.28)	(*p* < 0.01)	(*p* = 0.79)
Aorta-VOI-2	0.914	−0.076 ± 0.033	0.889	−0.047 ± 0.050
	(*p* < 0.01)	(*p* < 0.05)	(*p* < 0.01)	(*p* = 0.37)
Aorta-VOI-3	0.858	0.071 ± 0.061	0.832	0.143 ± 0.091
	(*p* < 0.01)	(*p* = 0.27)	(*p* < 0.01)	(*p* = 0.14)
Aorta-VOI-4	0.882	0.066 ± 0.057	0.848	0.142 ± 0.088
	(*p* < 0.01)	(*p* = 0.26)	(*p* < 0.01)	(*p* = 0.13)
Ventricle-VOI	0.819	0.141 ± 0.088	0.904	0.044 ± 0.046
	(*p* < 0.01)	(*p* = 0.13)	(*p* < 0.01)	(*p* = 0.35)

K* = hepatic systemic clearance of ^18^F-FDGal (ml blood/ml liver tissue/min) estimated using image-derived input function; *K* = hepatic systemic clearance of ^18^F-FDGal (ml blood/ml liver tissue/min) estimated using time-activity curve from manual blood sampling from the radial artery; VOI = volume of interest (see Figure 1 for details on each VOI definition). *r* = Pearson correlation coefficient for correlation between individual pairs of K* and *K*; *p* < 0.05 is considered to indicate statistically significant correlation. Relative deviation of K* from *K* is presented as mean ± SEM; mean deviation with *p* < 0.05 is considered statistically significantly different from zero.

**Table 2:** **Patients with cirrhosis (functional** ^**18**^ **F-FDGal PET/CT of the liver using image-derived non-invasive input function)**

**VOI**	**Without resolution modelling**		**With resolution modelling**	
	**Correlation between** ***K*** * and ***K*** **,** ***r***	**Relative deviation of** ***K*** * from ***K***	**Correlation between** ***K*** * and ***K*** **,** ***r***	**Relative deviation of** ***K*** * from ***K***
Aorta-VOI-1	0.911	0.059 ± 0.040	0.924	0.078 ± 0.039
	(*p* < 0.01)	(*p* = 0.16)	(*p* < 0.01)	(*p* = 0.07)
Aorta-VOI-2	0.904	0.044 ± 0.041	0.919	0.051 ± 0.037
	(*p* < 0.01)	(*p* = 0.37)	(*p* < 0.01)	(*p* = 0.19)
Aorta-VOI-3	0.897	0.162 ± 0.049	0.893	0.210 ± 0.052
	(*p* < 0.01)	(*p* < 0.01)	(*p* < 0.01)	(*p* < 0.01)
Aorta-VOI-4	0.874	0.169 ± 0.055	0.878	0.223 ± 0.057
	(*p* < 0.01)	(*p* < 0.01)	(*p* < 0.01)	(*p* < 0.01)
Ventricle-VOI	0.872	0.203 ± 0.058	0.895	0.173 ± 0.050
	(*p* < 0.01)	(*p* < 0.01)	(*p* < 0.01)	(*p* < 0.01)

K* = hepatic systemic clearance of ^18^F-FDGal (ml blood/ml liver tissue/min) estimated using image-derived input function; *K* = hepatic systemic clearance of ^18^F-FDGal (ml blood/ml liver tissue/min) estimated using time-activity curve from manual blood sampling from the radial artery; VOI = volume of interest (see Figure 1 for details on each VOI definition). *r* = Pearson correlation coefficient for correlation between individual pairs of K* and *K*; *p* < 0.05 is considered to indicate statistically significant correlation. Relative deviation of K* from *K* is presented as mean ± SEM; mean deviation with *p* < 0.05 is considered statistically significantly different from zero.

For healthy subjects, the mean relative deviation of K* from *K* was not significantly different from zero for any of the five VOIs or two reconstruction methods except for Aorta-VOI-2 without resolution modelling (*p* = 0.04) (Table 1). Based on mean relative deviation, Aorta-VOI-1, Aorta-VOI-3, and Aorta-VOI-4 performed equally well and were only slightly more accurate than Ventricle-VOI (Table 1).

For patients with cirrhosis, the mean relative deviation of K* from *K* was not significantly different from zero for Aorta-VOI-1 and Aorta-VOI-2 for any of the two reconstruction methods (*p* > 0.05) (Table 2). For Aorta-VOI-3, Aorta-VOI-4, and Ventricle-VOI, the mean relative deviation was significantly different from zero using both reconstruction methods (Table 2).

## Discussion

**Figure 2:**
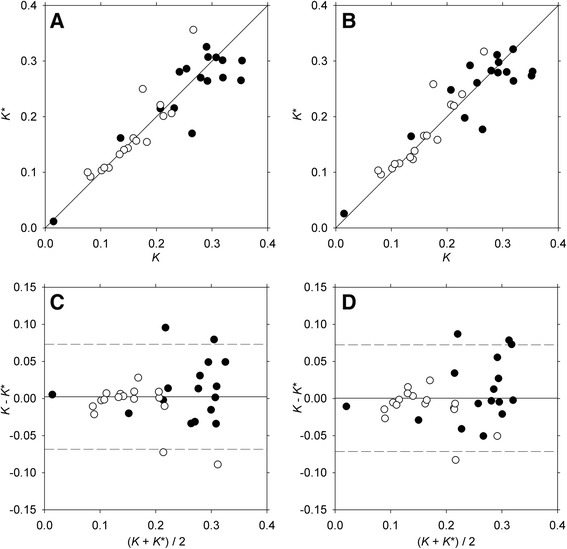
Correlation plots and Bland Altman plots for Aorta-VOI-1. Correlation between K* and *K* without resolution modelling **(A)** and with resolution modelling **(B)**. Bland Altman plot without resolution modelling **(C)** and with resolution modelling **(D)**. Closed circles (●) are healthy subjects, and open circles (○) are patients with cirrhosis.

This study demonstrates that arterial blood sampling can be successfully replaced by an image-derived TAC as input function when calculating metabolic liver function from dynamic ^18^F-FDGal PET/CT. All five image-derived input VOIs yielded K*-values that correlated with the reference value, i.e., *K* calculated using the Artery-TAC from arterial blood sampling but Aorta-VOI-1 was the only input VOI that yielded results not statistically different from the reference in both healthy subjects and patients with cirrhosis using both reconstruction methods. Figure 2A, B shows the correlation between K* and *K* for Aorta-VOI-1, and, as shown in the Bland Altman plots in Figure 2C, D, there were no systematic deviations for high or low values.

The healthy liver accumulates more ^18^F-FDGal than a cirrhotic liver [1,2], and the reason why Aorta-VOI-2 failed using the reconstruction without resolution modelling in healthy subjects may be due to spill-over from the liver in the anterior part of the aorta which was not included in Aorta-VOI-1 (Figure 1). This would make the input function too high, leading to an underestimation of K* (i.e., K* < *K*), which agrees with the observed deviation (Table 1). Since patients with cirrhosis have decreased metabolic liver function [1], the spill-over from the liver is expected to be lower which might explain why both reconstruction methods were successful for both Aorta-VOI-1 and Aorta-VOI-2 in patients with cirrhosis.

Both Aorta-VOI-3 and Aorta-VOI-4 yielded K* values that deviated significantly from *K* in patients with cirrhosis for both reconstruction methods. The close relation of these two VOIs to the edge of the aorta could explain this since most of the aorta is surrounded by structures with low radioactivity which could lead to an underestimation of the image-derived TAC when compared to the Artery-TAC. The calculated K* would accordingly be higher than *K*, which was true for all four cases (relative deviation > 0.162, Table 1). On the other hand, no significant deviation was present in healthy subjects. Spill-over from the *hot* liver might have increased the image-derived input in these VOIs, counter-acting the effect of surrounding structures with low radioactivity.

The Ventricle-VOI yielded K* values that were higher than *K*, but the difference was only statistically significant in patients with cirrhosis. The Ventricle-VOI is subject to both respiratory and cardiac movement as well as spill-over from the liver. However, in the present case, the overestimated K* values indicate inclusion of *cold* areas in the VOI (such as the myocardial wall and surrounding lung tissue) due to the respiratory and cardiac movements. We included the Ventricle-VOI in the present study because ventricle-VOIs sometimes are used to derive input concentrations in other studies, but in the present study, it failed to provide a good input function, probably due to the reasons discussed above. In addition, only the lower part of the left ventricle of the heart was included in the field of view because the main focus of the investigations was the liver. This may have contributed to a relative poor definition of the Ventricle-VOI.

An important potential application of dynamic ^18^F-FDGal PET/CT is creation of three-dimensional parametric images which makes it possible to study intrahepatic variation in metabolic capacity [1,2]. This can for example be used to spare well-functioning areas of the liver when treating liver tumours with stereotactic radiotherapy [4]. The simplification of the method by avoiding arterial catheterization makes it safer and more attractive to repeat over time when monitoring metabolic liver function in patients with cirrhosis, following different treatments of diseases of the liver, or for scientific purposes.

Because ^18^F-FDGal is very specifically taken up and accumulated in the liver, spill-over from other organs was not a major concern in the present case. However, for other tracers, such as the glucose tracer 2-[^18^F]fluoro-2-deoxy-D-glucose (^18^F-FDG) which is taken up in most tissues, another VOI definition may be required. One method for defining an image-derived input function for ^18^F-FDG of the liver was published some years ago with a circular Aorta-VOI drawn in the middle of the abdominal aorta for ^18^F-FDG PET of liver tumours using a dedicated PET camera [9]. It is recommended that an image-derived input function is tested individually for specific PET tracers and different reconstruction methods before proceeding to routine use of the method.

## Conclusions

We have defined an image-derived input function in terms of a semicircle drawn in the posterior part of the abdominal aorta that could replace arterial blood sampling successfully for quantification of metabolic liver function using ^18^F-FDGal PET/CT. This makes the method safer, simpler, and more generally applicable for routine clinical and scientific use.
